# Supplementary material for: The unified myofibrillar matrix for force generation in muscle
Source: Nat Commun. 2020 Jul 24;11:3722. doi: 10.1038/s41467-020-17579-6 (PMC7381600; doi:10.1038/s41467-020-17579-6)
Supplement: Supplementary file 1 — Supplementary Information [file 41467_2020_17579_MOESM1_ESM.pdf]

The Unified Myofibrillar Matrix for Force Generation in Muscle

Willingham et al.

Supplementary Information

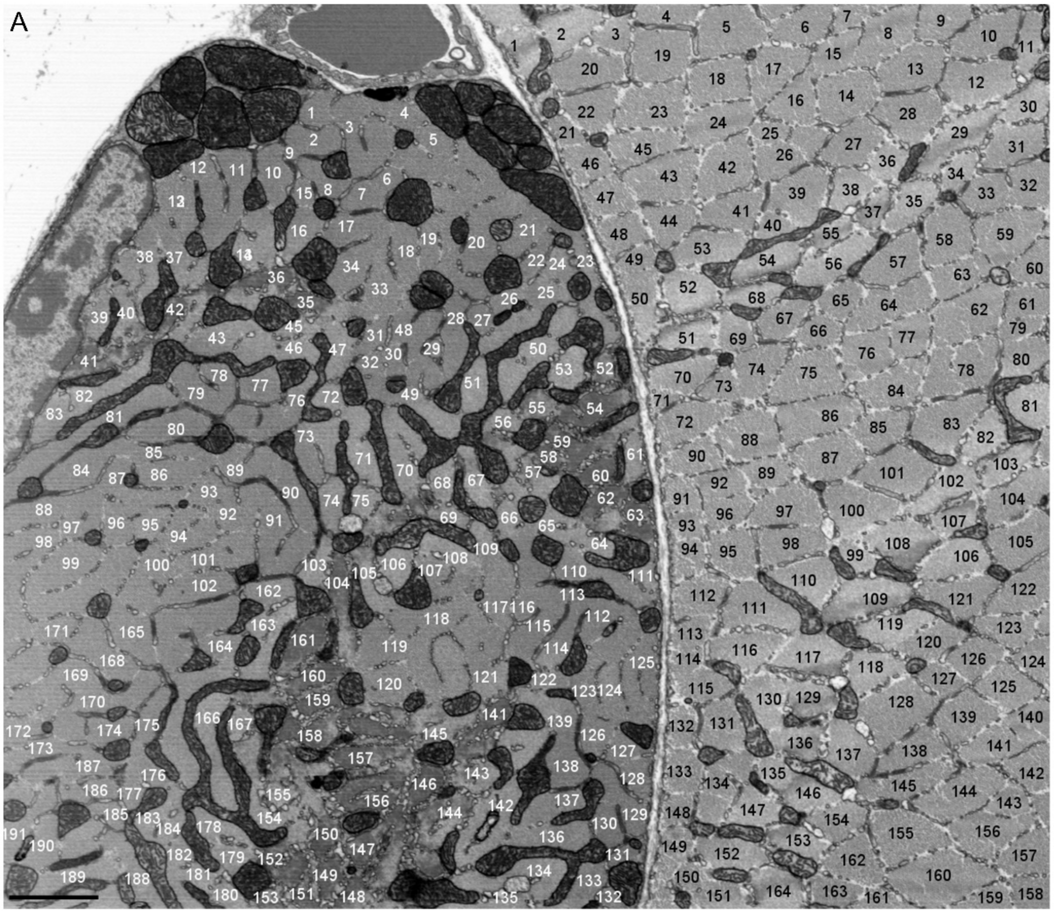

Supplementary Figure 1. Single FIB-SEM image showing adjacent fast- (right) and slow-twitch (left) muscle fibers and their many parallel sarcomeres (numbered). Image representative of 4 fast-twitch datasets and 3 slow-twitch datasets. Scale bar: 1 μm.

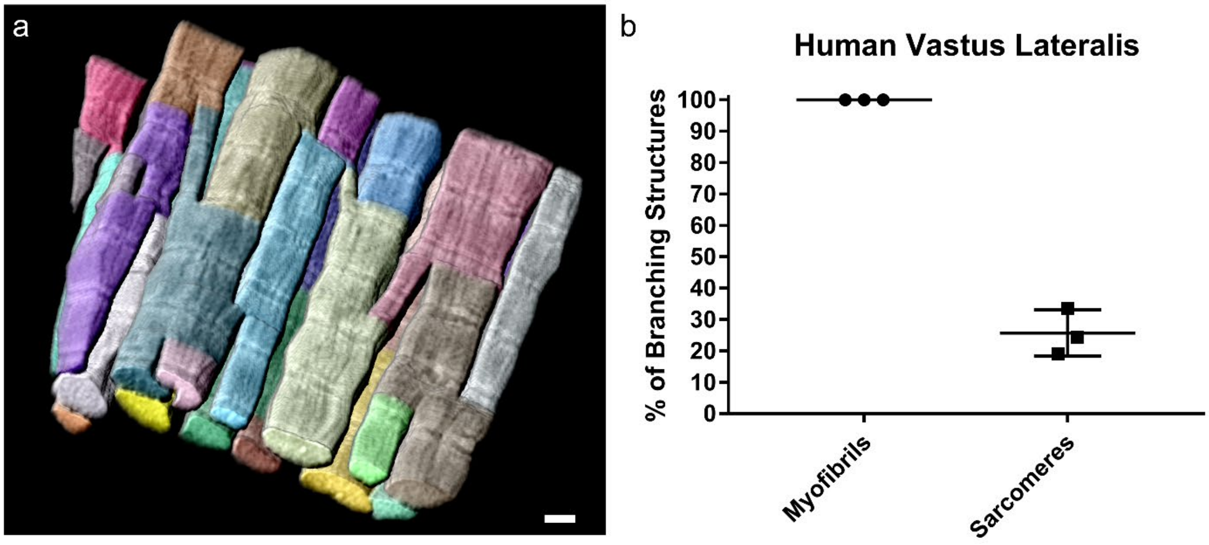

Supplementary Figure 2. a) 3D rendering of 34 connected myofibrillar segments (various colors) from human vastus lateralis muscle. Image is representative of 3 FIB-SEM muscle volumes. Scale bar: 1 µm. b) Percentage of myofibrils with at least one branch and percentage of sarcomeres with a branch in adult human vastus lateralis muscle. Raw FIB-SEM data with 30 nm pixel sizes obtained from the Baltimore Longitudinal Study of Aging[27]. Muscles were from humans less than 40 years of age. N=60 myofibrils and 906 sarcomeres assessed from 3 muscles. Bars: mean±SE.
