# Supplementary material for: The unified myofibrillar matrix for force generation in muscle
Source: Nat Commun. 2020 Jul 24;11:3722. doi: 10.1038/s41467-020-17579-6 (PMC7381600; doi:10.1038/s41467-020-17579-6)
Supplement: Supplementary file 11 — Description of Additional Supplementary Files [file 41467_2020_17579_MOESM11_ESM.pdf]

Title: Supplementary Movie 1.

Description: Raw image stacks (muscle cross-section on left, longitudinal view on right) from a FIB-SEM volume containing a fast- (right cell in cross-section) and slow-twitch (left cell in cross-section) muscle fiber followed by the structure of 115 directly connected sarcomeres within a region of the myofibrillar matrix of the fast-twitch fiber.

Title: Supplementary Movie 2.

Description: 360° rotation and zoom through of a 3D rendering of a region of the fast-twitch myofibrillar matrix.

Title: Supplementary Movie 3.

Description: 360° rotation of a 3D rendering of a region of the fast-twitch myofibrillar matrix and surrounding mitochondria (within 500 nm).

Title: Supplementary Movie 4

Description: 360° rotation of a 3D rendering of a region of the fast-twitch myofibrillar matrix and the surrounding sarcotubular system (within 200 nm).

Title: Supplementary Movie 5.

Description: Upper: 360° rotation of the z-disks (cyan) and sarcoplasmic reticulum (red, green, yellow) associated with the region of the myofibrillar matrix shown in Figure 1 and Supplementary Movie 2. Lower: 360° rotation of 3772 individual z-disks within a FIB-SEM volume of a fast-twitch muscle showing Vernier displacement of z-disk sheets.

Title: Supplementary Movie 6.

Description: Raw (left) and segmented (right) images from a FIB-SEM muscle volume showing an example of sarcomere splitting.

Title: Supplementary Movie 7.

Description: Cross-section (left) and longitudinal (right) views of a region of a FIB-SEM muscle volume showing an example of a myofilament transfer followed by 360° rotations of a 3D rendering of the sarcomeres involved in the myofilament transfer.

Title: Supplementary Movie 8.

Description: 360° rotation and zoom through of a 3D rendering of an entire 21 µm length of a muscle at postnatal day 1 showing the connectivity of the myofibrillar matrix throughout the cell. Individual myofibrillar segments: various colors, nuclei: cyan, cell membrane: green.

Title: Supplementary Movie 9.

Description: 360° rotation and zoom through of a 3D rendering of a minimally connected region within the slow-twitch myofibrillar matrix (grey) showing continuity across the width of the muscle fiber and

across myonuclear domains. Nuclei: cyan, cell membrane: green. Abrupt ends to myofibrillar structure represent connected regions not shown for visualization purposes.
